# Emerging Processes Within Peer-Support Hearing Voices Groups: A Qualitative Study in the Dutch Context

**DOI:** 10.3389/fpsyt.2021.647969

**Published:** 2021-04-21

**Authors:** Barbara Schaefer, Jenny Boumans, Jim van Os, Jaap van Weeghel

**Affiliations:** ^1^Parnassia Group Academy, Parnassia Psychiatric Institute, The Hague, Netherlands; ^2^Department of Community Care and Social Participation, Trimbos Institute – For Mental Health, Utrecht, Netherlands; ^3^Department of Psychiatry, Brain Centre Rudolf Magnus, University Medical Centre Utrecht, Utrecht, Netherlands; ^4^Department of Psychosis Studies, King's College London, King's Health Partners, Institute of Psychiatry, London, United Kingdom; ^5^Phrenos Center of Expertise for Severe Mental Illness, Utrecht, Netherlands; ^6^Tranzo Scientific Center for Care and Wellbeing, School of Social and Behavioural Sciences, Tilburg University, Tilburg, Netherlands

**Keywords:** hearing voices groups, peer support, self-help, auditory hallucinations, psychosis, personal recovery, qualitative research

## Abstract

**Purpose/Aims:** This study aimed to gain insight into the value of Hearing Voices Groups (HVGs) in the Dutch context. Specifically, we aimed to learn more about the meaning of HVG participation, as well as the aspects that contribute to that meaning, from the perspective of participants' experiences.

**Method:** The study used a qualitative design with in-depth interviews to explore the experiences of 30 members within seven HVGs in the Netherlands. Interviews were recorded, transcribed, and analyzed using interpretative analysis inspired by the Grounded Theory method.

**Findings:** The individual-level analysis revealed four different group processes that appear to determine the value that HVGs have for their participants: (i) peer-to-peer validation, (ii) exchanging information and sharing self-accumulated knowledge, (iii) connection and social support, and (iv) engaging in mutual self-reflection. We found that specific characteristics of HVGs facilitate these group processes and lead to specific personal outcomes. Combining the interview data from people who joined the same HVG reveals that, although all four described group processes occur in all groups, each group's emphasis differs. Three related factors are described: (i) the composition of the group, (ii) the style of the facilitators, and (iii) the interaction between group processes and individual processes.

**Implications:** Unique processes, for which there is little to no place within regular mental health care (MHC), occur within HVGs. MHC professionals should be more aware of the opportunities HVG can offer voice-hearers. Essential matters regarding the implementation of HVGs are discussed.

## Introduction

About 3–19% (median 13%) of the general population has heard one or more voices during their lifetime (1–3). Some people are not bothered by the voice(s) they hear and, sometimes, even see them as helpful (4, 5). However, some people suffer greatly from hearing voices. A relatively small proportion of voice-hearers seek professional help, which often consists of medication and cognitive behavioral therapy (6, 7). While these interventions may sometimes alleviate the experience, many voice-hearers indicate that they long for a place to express and make sense of their experiences in a safe and open environment (8, 9). In response to this need, Hearing Voices Groups (HVGs) provide such a place. In the last three decades, HVGs have become an established practice worldwide in both community and psychiatric settings (10).

The HVG approach is part of the Maastricht approach developed in the Netherlands by employees of the Social Psychiatry Department of the University of Maastricht in co-creation with voice-hearers who are both service users and non-service users (11). The Maastricht approach focuses on the importance of accepting voices and making sense of the whole voice-hearing experience (12–14). The approach has inspired many voice-hearers worldwide and has given rise to an international social movement, “The Hearing Voices Movement,” which offers an alternative to the traditional biomedical and cognitive-behavioral framework of hearing voices. For many voice-hearers, it was a relief to learn about this less stigmatized approach. The first HVG was established in the Netherlands in 1987, and the first HVG in England followed a year later (14). Hearing Voices Networks were formed in many countries, with the central tasks being the support, development, and operation of HVGs (15). The variety of settings where group meetings are held contributes to a healthy degree of diversity amongst HVGs while preserving core themes, which remain constant across international groups.

HVGs differ from regular psychoeducation and group therapy in that they have a focus on group ownership rather than following a predetermined, guideline-based structure (15). All HVG participants are considered experts due to their lived experience. Thus, no single expert exists, and all group members are experts. Members make decisions about how the group is run and what is talked about together. The HVGs provide a tool to help group members make sense of and cope with their voices on their own terms and in their own way through talking about their experiences and asking each other questions (15). Consistent with the Hearing Voices Movement's founding principles, diverse explanations are accepted for the origins of voices, and voice-hearers are encouraged to take ownership of their experiences and define them for themselves (9).

There has been growing scientific interest in HVGs in recent years. Given the Hearing Voices Movement's focus on group empowerment and the plurality of interpretation and expertise, evaluation through traditional evidence-based methods, with a focus on standardized treatment protocols, method “fidelity” and standardized outcome measures, cannot be readily envisaged (9, 16). Due in part to this difficulty, not many systematic studies about the impact of HVGs exist. Published work, mainly qualitative studies, suggests that participants get more out of attending HVGs than just the ability to speak freely about anything to do with hearing voices (17). Participants have indicated that they feel supported, develop coping strategies, improve their understanding of the voice-hearing experience, and develop better self-esteem and confidence (14, 18–23). However, most of these studies report on relatively small samples and often involve a select group of participants. One exception is a recently published American qualitative study, based on data from a large, diverse sample, that provides an important starting point for the theoretical understanding of how HVGs facilitate significant transformation through participation (24).

In the Netherlands, within the last decade, the healthcare landscape for people with severe mental illness has changed substantially. There is now an increased focus on recovery-oriented care and social inclusion (25, 26), although this has yet to meet with great success (27–30). The position of the HVGs within the Dutch healthcare landscape has not been much improved, and there are relatively few HVGs in the Netherlands; many mental healthcare professionals (MHC) find it difficult to refer people who hear voices to an HVG due to a lack of clarity about what “evidence-based” contributions these groups may make in terms of recovery and well-being.

This study aimed to gain insight into the value of HVGs in the Dutch context. Specifically, we aimed to learn more about the meaning of HVG participation, as well as the aspects that contribute to that meaning, from the perspective of participants' experiences.

## Materials and Methods

### Design

To gain insight into the value of Dutch HVGs we used a qualitative method, which consisted of open in-depth interviews with both existing and new members joining seven HVGs. The study was part of a larger study (conducted between January 2016 and December 2018) in which both quantitative and qualitative data were obtained. We used quantitative methods to explore how HVGs may contribute to the recovery process of people who hear voices (with questionnaires regarding, among others, their personal goals and whether they were met, their voices, empowerment, personal recovery and whether the group supported them in their recovery process). We used a qualitative method to investigate experiences with HVG participation. In this paper we present the results of the latter. Participants gave written informed consent regarding the entire study. We received an official exemption from the rigorous review by an MREC from the Medical Ethical Committee of the LUMC, because our study was considered as not being subject to the Medical Research Involving Human Subjects Act. A supervisory committee was established, consisting of researchers, board members of Stichting Weerklank (a Dutch foundation for and by voice-hearers) and Intervoice, and HVG facilitators who represented a mix of expertise by profession and by experience. The facilitators (in total three) facilitated three different HVGs and in that capacity they were present at the group member check session with facilitators. They were not involved in data-analysis to guarantee anonymity of participants and to avoid possible conflicts of interest.

### HVG Settings and Recruitment

Two board members of Stichting Weerklank, who were part of the study's supervisory committee, helped to approach HVGs to participate in our study. Almost all groups operating in the Netherlands around that time agreed. Unfortunately, several groups dissolved, mainly due to reorganizations within associated MHC organizations; however, new HVGs were also initiated. Ultimately, eight HVGs in the Netherlands participated in the study (see Table 1). The groups are well-spread across the country, some in urban areas and others in more rural settings. Almost all groups are associated in some way with an MHC organization or an organization offering supported and sheltered housing facilities. The five groups based at an MHC organization are only accessible to their own patients and participants meet in the same building where MHC treatment is given. The other three groups are open to anyone who hears voices and they meet at a location that is separated from MHC treatment facilities (e.g., a Hearing Voices Support Center). Facilitators in these three groups are all expert by experience. In the Netherlands there is not a specific training for HVG-facilitators. However, all groups had experienced facilitators who were knowledgeable about the ethos of the Hearing Voices Movement and often involved in this movement. Also, almost all the facilitators of the groups had completed a training of several days (“Understanding Hearing Voices” or “Coping with Hearing Voices”) that specifically focus on how to view and understand voice hearing in different ways, how one can cope with voice hearing and how to talk with voice-hearers about their voices. Some facilitators received a volunteer allowance and a few received nothing, but most were on the payroll of the organization to which the group is affiliated as a professional expert or an expert by experience. A total of 43 group members signed the informed consent for the study, of whom 30 could be interviewed. Since all members of one of the HVGs did not want to be interviewed, the data for this paper covers seven HVGs (see also Table 1, last column).

**Table 1:** Characteristics of the eight Dutch Hearing Voices Groups participating in the study.

**Group**	**Number of years active**	**Relation to Mental Health Care (MHC)**	**Facilitators**	**Frequency of meetings**	Approximate average number of group-members during the study^*^	**Number of participants interviewed**
1	5	Based at an MHC-organization	An experiential expert and a professional expert	2-weekly	7	3
2	4	Based at an MHC-organization	An experiential expert and a professional expert	2-weekly	7	4
3	3	No connection with an MHC-organization or Hearing Voices Support Center	No single facilitator	Monthly	5	3
4	1	Based at an MHC-organization	An experiential expert and a professional expert	2-weekly	12 (later eight, after a 2nd group–group five–was started)	10
5	1	Based at an MHC-organization	An experiential expert and a professional expert	2-weekly	7 (some were participants who came from group four)	2
6	1	Based at a Hearing Voices Support Center connected to an organization offering supported and sheltered housing facilities	Two experiential experts	Monthly	10	4
7	1	Based at a Hearing Voices Support Center connected to an organization offering supported and sheltered housing facilities	Two experiential experts	2-weekly	4	4
8	1	Based at an MHC-organization	One experiential expert	2-weekly	5	0

* *Almost all groups had a core of around four or five people with others participating more variably*.

### Data-Collection

A topic list for the in-depth interview was designed and discussed with the supervisory committee. Per the Grounded Theory interviewing method described by Charmaz (31), the interview consisted of three phases. In the first part, the participants were invited to tell about their experiences with HVG participation. The second part concerned (i) possible changes since joining the group, (ii) what they gained by attending their HVG, and (iii) what they liked or disliked about the group. The final part asked what participants found most important about attending a group and what they would recommend to facilitators starting a new group. The final question asked: “Is there anything we have not talked about that I should know to understand better what an HVG is about?”

The first author conducted all 30 interviews. Almost all were carried out within the usual group venue (one at the participant's home and one by telephone). The interviews took an average of 40 min (they ranged from 25–60 min) and were tape-recorded and transcribed (two participants did not agree to a recording but agreed to a summary of the interview). Participants were given the opportunity to read the report of their interview and to make additions where necessary. During data collection, a sample of the interviews was quality-controlled by the second author, who read the transcripts and checked for omissions and possible misinterpretations. Furthermore, the process of analysis already started during the end phase of data collection, allowing a (limited) degree of theoretical sampling to be applied.

We refrained from systematic observation within the group since the presence of a “researcher who comes to observe” (or doing a tape recording) could have influenced the meeting in question to the point of deviating from its natural course (32). However, opportunities naturally arose for the first author to attend several groups several times, which provided a better intuitive “feeling” of the environment. Whether the author could be present was always asked in advance to all group members. The author had to adhere to the group rules, so “what is said in the group remains in the group.” No notes have been made about these meetings.

### Analysis

We conducted an interpretative analysis inspired by the Grounded Theory method by Charmaz (31). We used the constant comparative method to discover patterns throughout the transcripts, to discern conceptual similarities and to refine the discriminative power of these categories (33). Through the process of categorizing, coding, delineating categories and connecting them, we were able to distinguish several concepts, themes, and processes that are important in understanding the value of HVGs for participants. All analyses were done on the Dutch transcripts (the quotes in the results have been translated for this paper into English). The first round of data analysis was focused on analyzing the transcripts of the individual participants. The first author selected 10 interviews, and the first two authors carefully read and reread these transcripts, separately identifying relevant concepts through a process of open coding. Software for qualitative analysis (MAXQDA) was used for this initial round, facilitating the coding process. Emerging concepts and conceptual categories were shared between the first two authors and discussed along with memos about observations and insights, resulting in a global coding frame. The first author repeated this procedure with the remainder of the transcripts, with which the initial coding frame was supplemented, tested, and expanded. The first and second authors then discussed possible connections between concepts (axial coding) and verified them against the data, resulting in a final theoretical model based on four relevant and internally connected group processes. In order to finalize the theoretical model, the first author conducted member checks with four participants of three different groups. The model and short descriptions of the different group processes were also shared and discussed within three HVGs. The basis of the model was very well-recognized. Adjustments were mainly made in the use of language (we have adapted certain terms to better reflect the experiences of the participants). After analyzing the individual transcripts, data were clustered and analyzed again at the level of the different HVGs. The same method of comparison was applied, however now explicitly focused on comparing segments from different members of one HVG. With the second author present, the first author conducted a group member check with the participating groups' facilitators (eight facilitators from six groups) to reflect on, especially, three group level findings. It was highly recognized that groups develop in different ways, and that, in addition to who participates in the group, this also has to do with the style of the facilitator and the need to reflect more and/or in more specific ways on what is being said in the group.

## Results

### Characteristics of the Participants

The group participants were quite diverse in terms of age, the age at which they first started to hear voices, the duration of voice-hearing, and the burden they experienced due to hearing voices at the beginning of the study (see Table 2). Per the Hearing Voices Movement ethos, diagnoses were not recorded, but we did ask some questions about contact with MHC, and this confirmed that it was a heterogeneous group of participants. Over a quarter of participants had been attending an HVG for over 18 months and some for 3 years or more.

**Table 2:** Characteristics of the participants (*n* = 30).

**Characteristic**	**%**
**Gender**	
Male	50%
Female	50%
**Age at the beginning of the study**	
20–29	3%
30–39	37%
40–49	30%
50–59	17%
60–69	10%
70–79	3%
**Age at first experiences with hearing voice(s)**	
0–9	20%
10–19	13%
20–29	34%
30–39	30%
40–49	3%
**Number of years of hearing voices**	
0–9	30%
10–19	30%
20–29	3%
30–39	20%
40–49	10%
50–59	7%
Burden of voice(s) at beginning of the study^*^	
1–2	10%
3–4	13%
5–6	17%
7–8	37%
9–10	23%
Age at first contact with mental health care^**^	
0–9	3%
10–19	25%
20–29	32%
30–39	29%
40 and older	11%
**Admitted to a clinic six months before completing the first**	
questionnaire^***^	
Yes	24%
no	76%
Contact with Mental Health Care in the past six months^***^	
Psychiatrist and psychologist and/or Social Psychiatric Nurse	55%
Psychologist and/or Social Psychiatric Nurse	24%
Psychiatrist (and occasionally another professional)	14%
Other (no contact or only the general practitioner)	7%
How do you rate the Mental Health Care that you got in the past six months^***^	
Bad	3%
Moderate	28%
Good	45%
Very good	10%
Excellent	14%
Medication in the past six months^***^	
No medication	11%
Antidepressants and/or tranquilizers	3%
Antipsychotic medication	61%
Antipsychotic and antidepressants and/or tranquilizers	25%

* *Item 17, “Intensity of distress” rated on a scale from 1 (not at all) to 10 (extremely), of the AVHRS-Q (34)*.

** *n = 28*,

*** *n = 29*.

### Findings Individual-Level Analysis

Although it was not always easy for interviewees to put into words exactly what they gained by attending an HVG, a vast majority indicated that the group was (or had been) especially important and that a participant “gains something” from attending the group. Many participants expressed sentiments like “It just gives me a very good feeling to join the group,” and they talked about feelings of relief, hope, inspiration, and belonging. They also talked about getting acquainted with new coping strategies and new ways to interpret their voices. Although the experiences were as diverse as the people who were interviewed, the analysis showed that personal experiences with the group were consistently attributed to several group processes that took place in the context of their HVG.

Four different group processes emerged in the analysis, which appeared to determine the value that HVGs had for their participants: (i) peer-to-peer validation, (ii) exchanging information and sharing self-accumulated knowledge, (iii) connection and social support, and (iv) engaging in mutual self-reflection. Our analysis found that the characteristics of HVGs help facilitate these group processes, which in turn lead to specific personal outcomes. The characteristics of each group process are further explained below.

#### Peer-to-Peer Validation

The first group process that emerged in the analysis arises from the fact that the HVG is a place where people can meet peers in a safe and “non-clinical” setting. For some, merely becoming aware that the group existed felt like a revelation, even before they met the other group members. Before they found out about the group, they did not realize that other people who heard voices might exist: this made them feel “different” and “alone.” Feelings of alienation and shame were profoundly rooted within many participants, with the result that talking about voices with other people was not something that came quickly. Awareness of the existence of HVGs opened a new world of opportunity for the participants: this was an opportunity to break the silence they had found themselves living with for years. Taking the step to join was sometimes a nerve-racking process, even though they knew the other members were also hearing voices; however, beyond the nervousness experienced during their first moments in the group, participants started to discover that the group's non-judgmental character helped them to talk freely about voices and other difficult topics. Meeting other people who hear voices gave participants tangible confirmation that such experiences exist and that others may experience the voices in similar ways. This validation process encouraged enlightening, liberating, and reassuring thoughts for many participants.

*I saw it [a poster about an HVG] on the bulletin board. A nice coincidence… Then I told my carers in the ward, and they said, “Yes, you should join.” And then… I was so happy… because before that time I thought… I didn't know that there were other people who… you know… Yes, that was so nice that I could participate… The first time I thought, “Ooh, I'm not alone!”*.

*You are not laughed at…. You can talk about anything*.

An essential feature of the HVG is that others' willingness to talk about their voices and other personal experiences has a contagious effect that allows them to overcome shame and hesitation. It appears that many participants shared experiences in the HVG that they had never told others. The ability to talk about specific things related to hearing voices without fearing possible negative consequences is often relieving and sometimes makes participants worry less and diminishes negative thoughts for a while.

*I find it a huge enrichment in my life that there are people who dare to talk so openly about their voices. That also gives me the courage to be open and honest about it*.

Very often, there was a lot of recognition or something…. Yes, that alone is nice… I feel. And… You know that others also go through something similar, and uh… Somehow that also removes somewhat… some fear of it…

The validating process of the HVG is not merely due to others experiencing the same thing, which decreases perceived stigma, but also because other participants understand what it is like to hear voices. Family, friends, and MHC professionals often try to understand what it is like to hear voices from a cognitive perspective but never from an experiential one. Group members not only know the experience of hearing voices but also easily understand what it is like to feel powerless when dealing with voices or what it is like to have to deal with misunderstandings of people who do not hear voices. Group members often recognize and accept the ambivalent, confusing, or erratic aspects of dealing with voices. This acceptance provides an opportunity to talk in more detail about things that are often left unspoken.

*So, the hearing voices group is mainly… meeting each other and talking about the voices, right… That is something special for many people, for me too. That you can just talk about it with others… outside of this group, that's hardly possible. I told a friend about the voices… but he doesn't really understand. These people [in the HVG group] know what it is, and so…. that makes a big difference. I've had many therapists, and they couldn't handle voices. They didn't know what to do about it and were not interested. Sometimes we tried… but that [their methods] didn't work out at all… So, you remain lonely and alone in that sense… yeah. This [the group] helps a bit. It is not very frequent but… just knowing that they are there… I look forward to going there*.

A vital characteristic of the group, which seems to enable validation processes, is that everybody takes each other seriously. Many perspectives on the experiences are accepted, and participants are not told they are “wrong” for having their own ideas regarding the origins of their voices. Furthermore, the groups are not introduced as therapy groups in which professionals give strict directions on what to do or think. Participants and the facilitators share their thoughts on all kinds of experiences regarding the voices, and each participant feels free to do whatever he or she wants to do with it.

*Yes, it is indeed striking how everyone puts their own twist on their voices. Some people give it a spiritual interpretation… some look for scientific explanations and then everything else in between. I believe that one of those people thought they honestly had contact with spirits, for example. It is just genuinely lovely… that all ideas are accepted. A facilitator must take everyone's views or opinions seriously. It means that everyone feels heard*.

#### Exchanging Information and Sharing Self-Accumulated Knowledge

Whereas, the first process within the group is related to the fact that HVGs make it possible to openly discuss hearing voices, the second process concerns the discussion content. For many participants, the HVG functions as a rich source of information to learn about how others experience hearing voices and how they deal with it. Of course, they could get their information about voice-hearing from books, the internet, or an MHC professional, but participants feel that receiving information live from peers has added value, primarily because they can learn many often unique and creative strategies from the group. The idea that others also struggle, or have struggled, to cope with hearing voices seems to stimulate people to actively start to find new ways to cope and even try out strategies that initially feel strange to them. They hear that different coping strategies can help different voice-hearers (at different times in their lives), which provides hope for trying different strategies.

Well, I think there are tips and tricks that psychologists don't know about… that are beneficial for people who hear voices. Maybe not all… and if it works for me, it doesn't have to be that way for someone else. I think many things can also work, but they are not “by the book.”

The acquisition of information is crucial in the early phases of attending an HVG. However, for participants who have been dealing successfully with voices for a more extended period, it can become increasingly meaningful to share self-accumulated knowledge with others. Participants emphasize how important it is to help fellow participants and “give something back” to the group. Sharing advice is an integral part of that; however, participants are also often cautious about doing so. They do not want to impose anything on the others to give everyone enough space to find their own way through their (recovery) process.

There were also people in the group who were a little further along in their process of… uh dealing with voices and things and uh… the things they reveal and how they dealt with it and what they did about it… Things like that… Yes, I found some of that useful… I thought, “Ooh, I will try that too,” or… uh, “Ooh, maybe I can do it that way.”

*I know how to deal with my voices… Before then, it controlled my life for a long time… I was terrified of it… but luckily, it is no longer like that… So, I particularly like that you can share experiences… That you can help other people because what I have noticed is that the people in the group… they are not as far along as I am. I like it when I can share my experiences and see that it is helpful to other people… yes… I just think it is valuable… it is also nice to hear all those examples… because, of course, that is what we are looking for… what helps in which situations*.

According to participants, the added value of the information exchange within HVGs arises mainly because the information is always related to real-life situations that currently occur for them. Because they determine the topics for the meeting together, the content is relevant to most group members. That the exchange of information within HVGs is grounded in experiences does not mean that there is no place for professional and academic knowledge. According to participants, the group, for example, can also discuss aspects of psychoeducation learned from MHC, academic publications, or research videos. Some members find these additions valuable. For others, this kind of information feels distant or irrelevant, and they would prefer to restrict group sharing to personal experiences.

*Last time I didn't like the subject very much. It was about hearing voices, and then the facilitator had been looking up things on the internet, and I found this wasn't too relevant [it was about brain scans of people who hear voices]. I usually deal with the voices in a practical way… trying to find solutions… and then I want to have tips [for dealing with them practically]*.

#### Connection and Social Support

The third group process is relational. The analysis shows that different interactions can arise between group members and result in different levels of connection. First, HVGs serve the basic human need for interpersonal contact, especially for people with a small social network. Just having social contact with others in the group, with whom they feel a certain kind of connection, prevents participants from feeling isolated and can be a healing experience in and of itself.

*It is one of those moments that I feel some connection with the rest of the world… Those moments are rare… If I didn't attend the group, I would communicate with anything and everything except people… and the chance of me no longer wanting anything to do with humanity would increase… But… my desire is really that I still want to be part of society*.

The second level is the emergence of mutual support, which is encouraged by the structure of the HVGs. Within the group, there is plenty of room to respond to each other and return to matters discussed earlier. It is also possible for participants to prepare for challenging situations together (e.g., family gatherings or job interviews) and evaluate their struggles and successes later. In this way, group members can celebrate new steps and small victories together.

What is clear in participants' stories is that based on togetherness and support, the bond between members of a group gradually grows and can lead to intense feelings of solidarity. This third level within the interaction process has to do with building trust. Real trust must grow: it develops not only because participants share their experiences but also because they are collectively responsible for their safety and the discussion topics. One of the few rules in HVGs is that what is said in the group remains confidential within the group. This agreement to mutual confidentiality can increase mutual trust, which is considered necessary to engender a more profound feeling of connectedness. That the groups do not enforce strict protocols allows group participants to veer naturally toward personal conversations to which many participants attach great value. People become more open about their thoughts and feelings and, thus, they get to know each other better.

If the group is stable and members can go through a “learning process” together, social connections can become more intense. These deep involvement processes do not always happen, but participants report that, when it does, it leads them to feel more valued and accepted for who they are, in addition to creating a sense of belonging. When participants know more about each other, mutual understanding and respect grow, and participants can ask more specific questions and give specific “feedback” to each other. These interactions help participants find what works for them when dealing with voices, positively affects self-confidence and resilience, and gives members the courage to express themselves fully. For some participants, the group restores humanity's confidence and helps them reinvest in real connections with other people. By finding “buddies” within the group, with whom they can learn to apply specific social skills, they can also build up courage in the context of social situations outside the group.

*We are a small group, but everyone always comes. We always hug each other when we see each other again. I hardly do that with anyone… and I don't think they do that with many people. So… we really care about each other… We just have excellent relationships. This was not the case initially, but this feeling of togetherness began [to emerge] after a year or so. I am moved by the people, the stories they tell… I am not easily moved by others… I always keep my distance. but that is not the case here… I am interested. I listen to them very carefully… and respond… It's just very nice. Almost a bit intimate*.

Finally, it appears that connectedness extends beyond the group for some participants; for example, when they develop friendships with each other and start meeting outside the group for a walk, a drink, or to experience a shared hobby. HVGs are open groups, and participants can attend the groups as long as they like, which allows participants to invest in each other. Some people who have left the group say that they find it a bit unfortunate because they have become attached to the group members. It is reassuring to them that they can always attend the group whenever they want.

*In the hearing voices group, I have many people with whom I could be friends. There are a couple of people with whom I would like to be friends. I often have people around who take care of me, and that… for me… is not friendship… I think… I find friendship is about equality… and that you can make jokes together*.

#### Engaging in Mutual Self-Reflection

The fourth and final process, which emerged during the analysis, concerns the reflection processes that arise when members ask each other sincere and open follow-up questions to gain more insight about their experiences. Here, too, different degrees of depth are distinguished. Almost all participants describe that talking about various topics related to hearing voices results in a certain degree of awareness. The HVG facilitates reflection about what the participants hear, what they think, feel, and do when they hear voices, and how they view the phenomenon of hearing voices. Other participants and facilitators encourage this process by asking specific questions tailored to the individual who is sharing. This kind of further probing emerges not from efforts to cure the participant but from a natural curiosity about how the other group members experience the voices they hear.

First, we go around the table so everyone can share how they feel. I like that… Yes… Because then you can… Then you can hear yourself talking, and that is a good thing… You can think… “Ooh, yes, yes… that's what I am doing.”

Trying to answer questions from other group members, listening to their stories, and asking the other members questions helps them reflect on their experiences and perspectives. Becoming more aware of how the voices influence their lives, about possible triggers, about the meaning of hearing voices, and how they relate to the voices helps participants to challenge beliefs that may stand in the way of healing on a personal or even existential level. Participants can explore alternative ways to view or approach certain situations. By reflecting together and exchanging ideas and theories, participants gain new insights. As a result, participants may alter their perceptions about hearing voices and how they interpret voices; they might begin to take actions that originate from a personal reflection process instead of trying a coping strategy just because someone says it might work.

*I have noticed that how I view the voice-hearing myself… that it is really something of mine… I think of it as… “My voice is my partner, and we have to work it out together.” If I have stories about this relationship, then I think, “the others will probably have that too,” but the others don't have that at all. They deal with it in a completely different way. Because of the other stories, I can also look at it from a different angle and do something with it or not. It keeps you focused on other possibilities*.

*I just love learning how it works for everyone. This way, I can gain insight into certain things. I have always thought… hearing voices… it's a short circuit in your brain, or it has to do with drugs… one of the two… And drugs can also cause a short circuit in the brain… so yeah… it's related. Only, I also know that there are people in the group who do not use drugs and that it probably results from tension and stress and all that… I heard from someone that the voices came from an antenna or satellite, and another said that they came from the television, and another said that it was the voice of God… I came into my apartment after that meeting, and I thought, “Yes… I will not let myself be fooled anymore.” I always resisted the voices… I defended myself… and from that day on, I just ignored the voices… and within days, the voices were gone*.

Although, for some participants, the collaborative process of self-investigation and developing interpretative frameworks to gain more insight into voice-hearing forms the core value of HVGs, the need to explore and gain insight can vary between individual participants. Some participants prefer not to think about their voices' significance; they do not find related questions or hypotheses worthwhile, given their own experiences. Some members cannot or do not want to reflect extensively and prefer to restrict their participation to sharing and hearing experiences.

### Findings Group-Level Analysis

Combining interview data of persons who participated in the same HVG revealed that, although all four described group processes occur in all groups, each group's emphasis differs. Three related factors include: (i) the composition of the group, (ii) the style of the facilitators, and (iii) the interaction between group processes and individual processes.

#### Influence of Who Joins the Group

**Figure 1:**
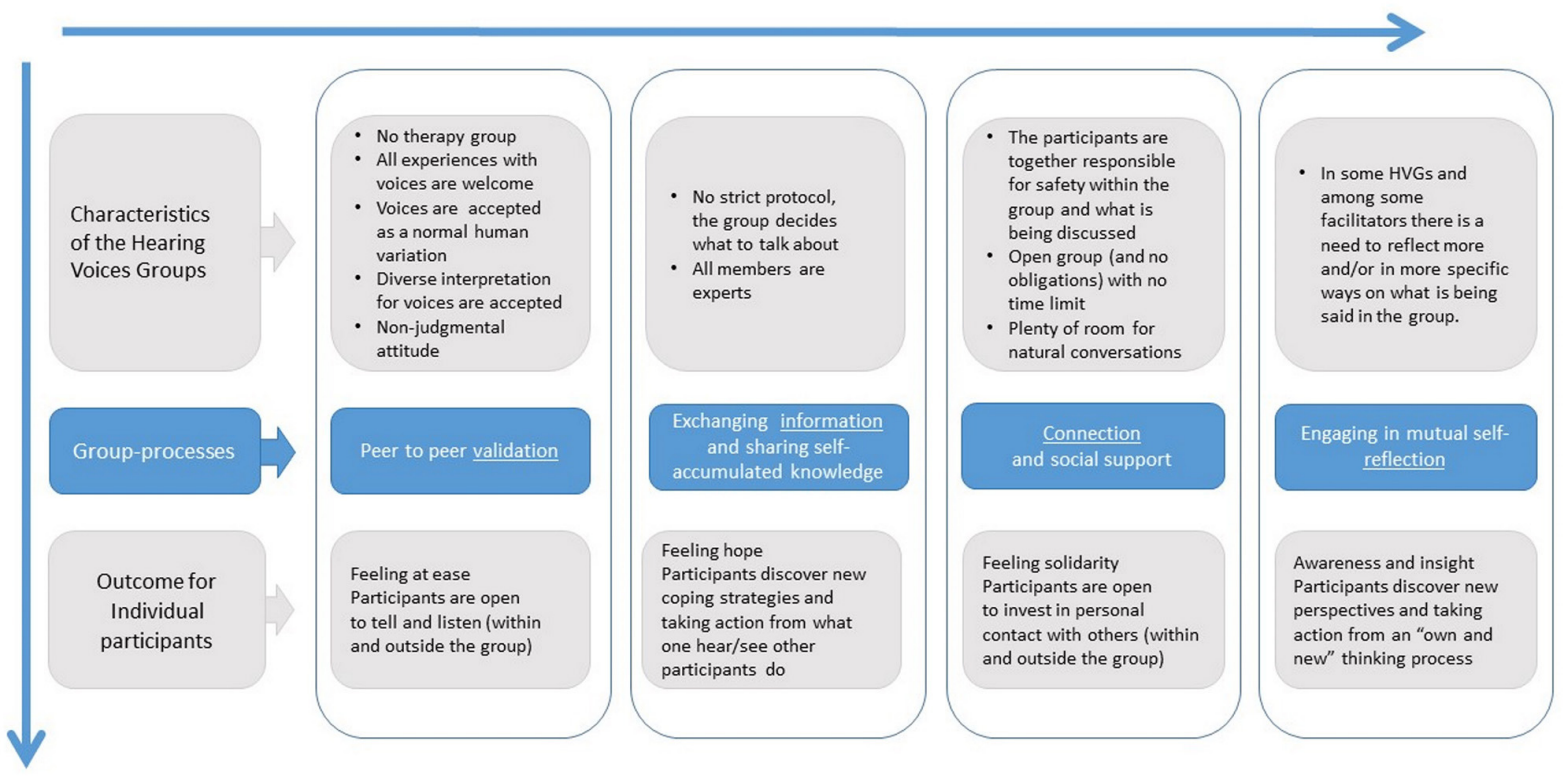
A model illustrating the four group processes that occur in Dutch Hearing Voices Groups. The characteristics of the Hearing Voices Groups help facilitate these group-processes, which in turn lead to specific personal outcomes.

The analysis shows that, for new groups or those that include participants in the “startling” or early “organization” phases of their recovery, during which they are still overwhelmed by hearing voices or have just started to begin coping (11), the group focuses primarily on *validation* and sharing *information* (the first two columns of the model, see Figure 1). In these groups, most participants are mainly concerned with actions that come from what they hear and see others do; for example, gaining the courage to talk more about personal experiences or trying different coping strategies to better deal with hearing voices. The effects of validation and information reception—feeling recognized and relaxed, experiencing relief and hope, and becoming more open to listening and sharing personal experiences—ultimately helps the participants to think more effectively about their experiences and the options available to manage them. In these groups, *connection* and *reflection* (the last two columns of the model, see Figure 1) also develop, but these tend to remain somewhat superficial.

Some groups move from validation processes and information toward more profound forms of connection and reflection, especially within groups with a stable composition and that have been meeting for some time. A shift to more mutual self-reflection often occurs when groups mainly include people less overwhelmed by their voices and further along in the “phase of organization” concerned with giving meaning to and trying to understand their voices (11). Participants then share a desire to change how they cope with the voices and are more interested in openly exploring their thoughts, feelings, and actions together. A safe place is essential for participants to explore their voices more thoroughly and deal with the questions that arise during this process: feelings of connection help create this space. Participants must feel confident in themselves and the group before investigating and taking ownership of their voices. When participants feel safe enough to explore these processes together with the group, they become involved in each other's “learning processes” and describe that it feels they are working together, even though they also feel empowered to move forward independently.

Although connection processes and mutual self-reflection benefit from a certain degree of similarity in participants' phases of recovery and stable group composition, it is not always possible to predict whether a group will, in fact, progress in a certain way. For example, perhaps some variation in membership and new input in the group creates a new challenge. Participants (and facilitators in the group member check) describe that it is sometimes just waiting for the right moment to hear something new (or something again but in a different manner and at the right moment) that set new things in motion. In the meantime, the group can serve as a place to share personal experiences and get support.

*The atmosphere in the group felt very fragile for a long time… People were hesitant… and then suddenly the subject of sexual assault or rape came up, and it turned out that quite a few people had experienced that… and as soon as one had said that… yes… then the others were no longer embarrassed about it and then… people started to reveal more things… uh… things they did not share before… Yes… that moment changed something in the group… But on the other hand… look… it is a small group… so what happens in the group depends very much on who is present… The way they talk… I have left the group now… And someone else may join…, and someone else may leave, and then the whole atmosphere might change again*.

#### Influence of Facilitators

The facilitator can also influence developments within the group. All facilitators seem to create a “free space,” where every participant has an opportunity to devise unique coping strategies; however, some facilitators seem to have a more directive style than others. Some facilitators reflect more or in more specific ways on what is being said in the group. Some seem to embrace Romme and Escher's Maastricht approach, where the aim is to accept the voices and find the connection between the voices and the intense event in someone's life that brought them out of emotional balance. Other facilitators talk about this approach but do not discuss voices from this perspective more than any other perspective. The group member check with facilitators confirmed style differences, but all reported that they find it crucial that facilitators know different ways to look at voices and different coping strategies.

Although every facilitator brings unique qualities to the table and therefore cannot easily be assigned to a category, it is apparent that, in groups facilitated by experiential rather than professional experts, the participants often have and take more freedom to decide what they want to talk about. These facilitators tend to remain in the background or function as a group member who also happens to arrange the location and ensure that group rules are followed. Conversely, professional experts often have a more directive style: either they or the group expect such an approach, especially when the group members are less emancipated and expect that these professionals take charge. These facilitators often take the lead and, although they respect group rules about non-judgment, the plurality of interpretations, and participant-directed discussion, they are often more focused on leading the dialogue in particular ways or a specific direction. These differences in facilitation styles influence the different group processes, mainly related to information-sharing and mutual self-reflection.

*Our facilitator is only partially a facilitator… [our facilitator is] especially practical… sends the mail out, arrange the location and coffee… so that all goes perfectly… During the meeting, our facilitator says, “Yes, now it is time to…” Again, our facilitator does not intervene much… but that doesn't matter because we don't really need it. We do that ourselves… It has grown like that. At first, I thought,… you need a strong moderator… but no… our facilitator also has experience with voices and is just a member of our group*.

*An experiential expert understands well what voices are, and they can also tell you how they did things themselves. Uh… but a professional expert is good at bringing up themes… is good at… well, structuring… And they know a lot about symptoms and can immediately tell you…what could be the cause and whether it is normal… I notice from the group… I notice myself… I feel more secure when there is a professional expert present [than only an experiential expert]*.

#### “Mismatch” Between a Group and an Individual Participant

The third finding reveals something about the way that group processes and personal processes interact with each other. We saw many examples of such fruitful, inspiring, and constructive interactions in the data, which led to positive outcomes. However, if an individual's focus is significantly different from the group's focus, this has consequences for the person involved. Such differences are not necessarily harmful and can undoubtedly lead to growth, but sometimes it is a reason for a person to leave the group (temporarily) because they experience no or insufficient positive effect (anymore). The data reveals some examples of a “mismatch” between a participant and the group, which can be traced back to three main issues.

The first concerns the Hearing Voices Movement's ethos, which describes hearing voices as a normal human variation and emphasizes that hearing voices does not necessarily indicate the presence of disease (9). This vision helps many participants adjust their self-image and challenge the societal idea that people who hear voices must be mentally unstable, like a ticking time bomb. However, some participants attach great importance to their diagnosis and find it difficult *not* to see their voice-hearing as a disease. They sometimes find it challenging to deal with others' positive recovery stories because they have long felt that they have little influence over their voices and find it difficult to take ownership of them. This view can cause restlessness and may even lead them to quit the group temporarily or maybe permanently.

The second reason for a possible mismatch occurs when a participant wants to discuss specific themes that differ from the group's preferred focus. Sometimes, hearing the experiences of others can cause excessive tension. For example, a participant may become overwhelmed by hearing about numerous potential explanations for the voices that they have not considered previously. Likewise, a participant who has just started managing the voices with medication may feel uncomfortable during discussions about tapering medications. At other times, a participant may wish to delve deeper into the voices' significance, but other group members are hesitant to do so. Sometimes participants do not wish to discuss the topic at hand or cannot relate to it. Of course, groups are typically sensitive to these issues, but it can be challenging to choose themes and topics that serve everyone. If the needs of all involved do not align, mainly concerning personal reflection, a participant may decide to stop coming to the group meetings.

Finally, a mismatch can arise based on the voices' tone or nature. For example, if only one participant hears positive voices while others suffer from malevolent voices, it can be difficult for them to relate to each other. Age is also a factor, and being the only young adult among a group of older adults can undermine feelings of belonging. Such misalignments prevent these individuals from taking full advantage of the processes of validation, information, connection, and reflection within the group, which means that they regularly leave the group after a while. Often, however, they feel that HVGs, overall, have the potential to meet their needs and, therefore, pursue membership in another HVG, or even the same one, at a later date.

*Some stories are so unrealistic… delusional… psychotic… and then I think, “What are you doing here?… go solve that first.” But of course,… you can't say that, because everyone has the right to attend the group and speak from their own perspective… and I respect that. So… but… you are not on the same level in terms of thinking and reasoning… I think… those differences within the group are too significant… If you smooth that out a bit… You can get… uh… much further within each group*.

*Most of the participants only heard negative things… I was the only one with solely positive voices… I also like to talk about it… I'm not ashamed of it. The others in the group said, “Don't argue with the voices, because then they'll get angry,” but, with me, the voices have never been angry… Most people want to get rid of the voices… and I don't mind them… No, for me, the HVG is of no use anymore… I experience hearing voices as a positive thing, and I haven't met anyone who experiences it positively yet*.

## Discussion

This study aimed to contribute to an understanding of the value of HVGs in the Netherlands. The findings illustrate how particular characteristics of HVGs facilitate four different group processes, which result in different individual outcomes. These characteristics, processes, and outcomes describe the core, unique values of the (Dutch) HVGs. Participants appreciate their HVG, not least because they know of few other places where such processes take place in this way. HVGs offer them new opportunities and meet individual needs that have not been realized, or have been realized only to a limited extent, within professional MHC or elsewhere in society, in particular the need to simply talk openly about hearing voices, and the need for exploring (the meaning of) the voices. Although similar group processes may take place in other peer-based interventions [e.g., recovery groups like Wellness Recovery Action Planning (WRAP); (35)], it seems that the specific characteristics of HVGs—no attendance limits or obligations, group ownership, and the acceptance of various perspectives on the origin and meaning of the voices heard—optimize these processes and create an ideal context in which voice-hearers benefit in different ways.

Our results support previous global research [see (17)], suggesting that HVGs contribute positively to participants' recovery process, for example, by allowing them to learn new coping strategies, develop social connections, and feel a sense of agency. The features that participants value, for example, non-judgmental atmospheres, a plurality of interpretation, and group-led discussion, further contributes to these positive outcomes. Our study enriches previous findings by highlighting possible connections between concepts such as HVG characteristics, processes within the group, processes within the members, and personal outcomes. Hornstein et al. (24) have recently undertaken a similar approach. Our data supported the processes found in Hornstein's study, but because the theoretical models of both studies have different starting points, we were able to add new findings. Perhaps the most significant contribution of this study is the awareness that, although valuable processes arise within HVGs, it is not guaranteed that each participant will embrace or progress through these processes to the same degree. Our results show that the match between the group and the individual participant plays an essential role in an individual's optimization of HVG meetings and that some participants may temporarily or permanently cease attending before significant changes have occurred.

Our findings suggest that all participants need validation and desire to investigate whether there is more information they can use to their advantage. All groups offered some help in this regard, with the result that all participants reported that they “really gained something” by attending their HVG. However, the participants' needs and potential for connection and reflection are diverse. The group processes related to these topics were more complex and layered than those related to validation and information. Our group-level analysis suggests that processes that concern connection and reflection take time to evolve within a group and may depend more on the group participants' personalities, including facilitators. Participants must sometimes be patient and willing to surrender to unfamiliar processes. For some participants, this is precisely what makes the group attractive to them, but others cannot wait for the group to engage with a topic important to them or are not ready to consider new perspectives.

### Clinical Implications

Unique processes, for which there is little to no place within regular MHC, occur within HVGs. The non-judgmental approach to voices in HVGs is often met with relief: voices are not immediately seen as a symptom of a disorder or mental disturbance, as this phenomenon is often interpreted by society and MHC. The natural, spontaneous, and dynamic processes of an HVG deviates from what people are used to in treatment groups in MHC, which often use strict protocols. Creating free spaces can be valuable to the recovery process because it helps people investigate their problems together, rather than alone, without the pressure of judgment and predetermined goals and strategies (36). MHC professionals should be more aware of the benefits HVGs offer to voice-hearers. They need to understand why processes like validation and sharing experiential knowledge are essential if only to know how this allows voice-hearers to feel more comfortable sharing their experiences; it was interesting to hear that many issues were not initially shared with MHC professionals, but were freely discussed in the group. HVGs also allow participants to connect naturally with their peers. This connection can reduce feelings of loneliness, which voice-hearers seeking professional help report as a problem (37, 38). Our results suggest, in accordance with other literature about HVGs (17) that HVGs can contribute significantly in specific ways to the well-being of voice-hearers. Recognizing these benefits could potentially lead to more referrals to HVGs.

Many of our study participants indicated that they experienced the groups as an enrichment of their treatment in MHC. They see the HVG as a valuable addition to their MHC treatment. Most study participants receive a lot of MHC and use medication. In the interviews, some participants of HVGs based in MHC indicated that many group members have significantly more problems than they do. These participants wanted to reduce their MHC, but also wanted to keep the option of joining an HVG. However, they cannot participate in their HVG once MHC-treatment stops. HVGs not related to MHC could be an option for them. These groups can play an important role in (relapse) prevention. They can support “ex-patients” in further personal and social recovery and they can offer accessible or alternative help to people who prefer not (yet) to contact MHC. There are still few of these groups, which means that no group is available in large parts of the country. Since the civil rights movements of reformist psychiatry in the 1970 (later framed as “antipsychiatry”) and the “recovery movement” in the 1980s, the patient's voice has gained influence in MHC and science. However, patient-driven progress had to be “conquered” over institutional resistance (39). With the advent of recovery colleges, user research centers, “multi-expert” eCommunities and many other examples of peer-support, which operate in parallel outside the MHC system, patient movements are increasingly empowered (40). In the Netherlands, for example, there are more and more recovery colleges who would like to start offering HVGs outside the MHC system. It is important that HVG facilitators are properly trained. Stichting Weerklank is considering further addition to the existing training courses in order to support HVG facilitators even better in their work.

Our study's results may also guide the implementation of HVGs, especially concerning the possibility of misalignments between the specific offerings of an HVG (at that time) and individual participants' needs (at that time). The first “mismatch” mentioned within the results section had to do with the ethos of the Hearing Voices Movement in describing hearing voices as a normal human variation. Some participants use predominantly “turning away narratives” described among others by de Jager et al. (41). These participants may have a hard time in an HVG when fellow group members want to explore the meaning, purpose, and origin of the voices together. Participants who predominantly “turn away” from their voices are often unwilling to challenge their voices or test their beliefs about them. They use whatever means are available to them to survive the experience and often seek a medical explanatory model that positions their voices as symptoms of a disease (41). Whether these participants stay with the group depends highly on what the other members want to discuss and whether they feel safe and accepted. Furthermore, the non-judgmental nature of a group and the view that multiple causes or significances of the voices are possible allows participants to see the phenomenon as a disease or not. The general idea behind the HVGs is that all voice hearers should feel welcome and be able to develop in their own way. However, one might also argue that another intervention that is more in line with a participant who wants to “turn away” from voices may be more appropriate for that individual.

The second reason for a possible mismatch concerns a participant's needs, especially concerning reflection, which the group cannot meet due to the participants being in different phases with regard to coping with their voices as described by Romme and Escher (11). Although our findings support Hornstein's (24) claim that “HVG can serve different functions at different times” and “variability in members' needs and levels of experience in the group are seen as strengths,” our data also shows that meeting all needs can be challenging at times. In an HVG, it is the task of the entire group to pay close attention to each other; however, the facilitator needs to be sensitive and responsive to an individual's needs while balancing those of the other group members. Additional individual guidance is sometimes very welcome, although it is sometimes also necessary for participants to be patient and accept a degree of uncertainty within the group. Not everyone can do so, and sometimes the differences are so significant that someone leaves the group regardless of everyone's best efforts. The establishment of more HVGs in the Netherlands would increase participants' opportunities to find a better match in this case. The same holds for the last issue mentioned in the results. Other countries have specific HVGs with, for example, young people or patients who reside in hospital care (15). It seems that such additions would be quite welcome in the Netherlands, given that some of our participants reported feeling that they did not quite “match” with their group but did see some potential in what HVGs offer.

### Strengths, Limitations, and Recommendations for Future Research

Our study is based on a heterogeneous sample of different groups' participants, which minimizes the probability of overlooking essential topics. In addition to study participants, we were also able to speak briefly with the group members (informal contact before or after group meetings) who did not want to participate in our study. We assume that their responses would have been similar to those captured by the study: most individuals who declined to participate wanted to talk about themselves and the group with the researcher but did not want to sign an informed consent and participate in an official capacity. However, we did not include individuals who attended the group only once or twice and then ceased coming to meetings.

In this study, we were for practical reasons not able to fully apply the Grounded Theory method, however following many of its analysis steps, we were able to give depth to the interpretation of the data. We entered the interviews with an open mind and attitude. We used a topic list in which the topics were not based on previous studies' themes or results. This approach allowed the participants to tell their stories without outside influence and express what was most important to them. The strength of our findings is that it portrays the subjective experience of participants.

For future research it would be instructive to see whether connections between concepts can be confirmed in other research, particularly quantitative studies, although it is challenging to evaluate concepts such as group characteristics and processes through standardized measurements. As became clear from the interviews, participants really appreciate having a place where they can share highly sensitive and traumatic experiences with others. In view of the literature on trauma and distressing voices (42, 43), it seems interesting to pay more attention to the role of sharing highly stressful experiences in future research on HVGs. It would also be useful to see how HVGs relate to other groups for voice-hearers, such as cognitive behavioral therapy groups; however, this also poses some challenges: our results show that HVGs are somewhat diverse, especially with regard to mutual self-reflection, and the founders of the HVGs indicate that randomly assigning people to HVGs does not fit well with the ethos of the groups where people really should have the freedom to join or not. Finally, comparing participants' mean group scores from HVGs to scores from structured and time-limited cognitive-behavioral therapy groups may not be a sufficiently comprehensive approach. Future research should focus more on what environments and strategies work for participants and whether these differ according to recovery stages and personal characteristics and preferences.

## Data Availability Statement

The datasets generated for this study are partly available upon request to the corresponding author.

## Ethics Statement

The studies involving human participants were reviewed and approved by Commissie Medische Ethiek of the LUMC. The participants provided their written informed consent to participate in this study.

## Author Contributions

BS: recruiting participants and performing the interviews. BS and JB: analysis and writing – original draft. JW and JO: writing – review and revising. All authors formulated the research questions and designed the study, contributed to the article, and approved the submitted version.

## Conflict of Interest

Board members of Stichting Weerklank and Intervoice members were part of the supervisory committee of this study. They contributed to the design of the study, the development of the topic list for the interview and to the recruitment of HVGs, but were not involved in data analysis to avoid conflict of interest. The conclusions in this paper are based on the structured interpretation process by the authors. The authors declare that the research was conducted in the absence of any commercial or financial relationships that could be construed as a potential conflict of interest.
